# Predicting inpatient hospital payments in the United States: a retrospective analysis

**DOI:** 10.1186/s12913-015-1040-8

**Published:** 2015-09-10

**Authors:** Mark W. Smith, Bernard Friedman, Zeynal Karaca, Herbert S. Wong

**Affiliations:** Truven Health Analytics, 7700 Old Georgetown Rd, Suite 650, Bethesda, MD 20814 USA; Agency for Healthcare Research and Quality, 540 Gaither Road, Rockville, MD 20850 USA

## Abstract

**Background:**

The Affordable Care Act (ACA) has increased rates of public and private health insurance in the United States. Increasing coverage could raise hospital revenue and reduce the need to shift costs to insured patients. The consequences of ACA on hospital revenues could be examined if payments were known for most hospitals in the United States. Actual payment data are considered confidential, however, and only charges are widely available. Payment-to-charge ratios (PCRs), which convert hospital charges to an estimated payment, have been estimated for hospitals in 10 states. Here we evaluated whether PCRs can be predicted for hospitals in states that do not provide detailed financial data.

**Methods:**

We predicted PCRs for 5 payer categories for over 1,000 community hospitals in 10 states as a function of state, market, hospital, and patient characteristics. Data sources included the Healthcare Cost and Utilization Project (HCUP) State Inpatient Databases, HCUP Hospital Market Structure file, Medicare Provider of Service file, and state information from several sources. We performed out-of-sample prediction to determine the magnitude of prediction errors by payer category.

**Results:**

Many individual, hospital, and state factors were significant predictors of PCRs. Root mean squared error of prediction ranged from 32 to over 100 % of the mean and varied considerably by which states were included or predicted. The cost-to-charge ratio (CCR) was highly correlated with PCRs for Medicare, Medicaid, and private insurance but not for self-pay or other insurance categories.

**Conclusions:**

Inpatient payments can be estimated with modest accuracy for community hospital stays funded by Medicare, Medicaid, and private insurance. They improve upon CCRs by allowing separate estimation by payer type. PCRs are currently the only approach to estimating fee-for-service payments for privately insured stays, which represent a sizable proportion of stays for individuals under age 65. Additional research is needed to improve the predictive accuracy of the models for all payers.

## Background

Three major elements of hospital cost accounting are the charge to payers, the total payment received from payers, and the cost to produce the services provided. Payments usually fall well below charges because of negotiated discounts and delayed or missed payments.

Under the Affordable Care Act (ACA), more than 20 million people are expected to gain insurance through Medicaid or health insurance exchanges [1]. Increasing coverage could raise hospital revenue and reduce the need to shift costs to insured patients. The consequences of ACA on hospital revenues could be examined if payments were known for most hospitals in the United States. Reliable payment data could also enable consumers to choose providers that offer better value than others, eventually leading to market-level gains in efficiency. Such data would also support research on how hospitals negotiate payments for specific conditions, how prices vary among payers, and the financial implications of payer mix within a hospital market area.

Medicare and Medicaid fee-for-service payments can be calculated based on publicly available information, although the effort is labor intensive. Payments from private payers are not publicly available because hospitals treat them as proprietary. A standard approach to estimating private payments has been to apply the Medicare cost-to-charge ratio (CCR) to charges for inpatient stays; if profit margins are low then on average costs should be similar to payments. This method is only approximate, however, in two senses: payments received are not the same as costs incurred, and there can be wide variation in the payments made by private payers, Medicare, and other payers for identical stays. Improving the quality of economic analyses will require better methods for estimating payments.

Levit, Friedman, and Wong [2] obtained confidential financial data on community hospital stays in 10 states. The data enabled them to calculate hospital-level “price-to-charge” ratios (PCRs) for five types of payers: Medicare, Medicaid, private insurance, uninsured individuals, and other payers such as workers compensation programs. (Because of the ambiguity of *price*, we will hereafter refer to PCRs as *payment-to-charge* ratios. The construction and meaning remain the same as in Levit et al.) The estimated payment for a stay in hospital *i* funded by payer *j* is simply the charge multiplied by PCR*ij*.

The purpose of PCRs is to enable researchers to readily estimate the payment for a hospital stay when exact payment data are unavailable. Data sources for the PCR estimates include patient demographic and clinical information from administrative claims data, as well as publicly available information on the hospital and its market, and selected state policies. The PCR is the link between charges, which are widely available, and payments, which are not. PCRs improve on the traditional CCR method by directly estimating the payment rather than approaching it indirectly through estimated cost and by providing better granularity in payer types.

Accuracy of the PCR is essential to reliable prediction of payment. Accurate PCRs would be assured if appropriate financial data were available for all hospital stays. At this time, however, only 10 states of the 46 contributing data to the Healthcare Cost and Utilization Project (HCUP) provide the requisite financial data. We therefore extended the work of Levit et al. [2] by investigating whether PCRs can be estimated for states in which such financial data are not available. Our approach was to model the PCRs in the 10 original states using only the information that is widely available for states contributing to HCUP. We then assessed the size and stability of out-of-sample prediction errors for each payer category and determined the correlation of the PCRs with traditional CCRs.

## Methods

### Empirical model

Our goal was to model a separate ratio of payments to charges for each payer. The data consisted of individual claims that were aggregated to stays and sorted by primary payer. The payment-to-charge ratio for payer type *j* is the ratio of total payments to total charges across all stays at the hospital with that primary payer. Nearly all values are positive and a few exceed 1.0. We retained for analysis stays associated with PCRs greater than zero and no greater than 1.0; the others represent outliers or cannot be modeled with a logarithmic link function.

We predicted the five PCRs in separate equations as functions of state, market, hospital, and patient characteristics using a generalized linear model with a log link and gamma-distributed errors. Each equation has this form:

$$ g\left(PC{R}_{ij}\right)={\alpha}_j+{\beta}_{ij}{C}_{ij}+{\gamma}_{ij}{H}_{ij}+{\delta}_{ij}{S}_i+{\varepsilon}_{ij}, $$

where *i* indexes hospitals, *j* indexes payer category, *C*_*ij*_ represents casemix variables, *H*_*ij*_ are hospital characteristics, and *S*_*i*_ are state characteristics that apply to every hospital in the state. A time subscript was unnecessary because we analyzed a single year of PCR data. The log-linear specification was chosen on the basis of skewness in residuals of ordinary least squares models. A PCR is specific to a payer *j* within a hospital *i*, so the unit of analysis was the hospital-payer combination. Person-level characteristics were therefore represented by hospital-level means across all stays in the dataset.

There is potential endogeneity among the PCRs at a hospital. In a study of the links among hospital market concentration, pricing, and profits, Robinson [3] found evidence that hospitals cut costs in response to lower Medicare margins, thereby improving margins for both publicly and privately insured patients. Hospitals may react to higher private-sector profits by raising costs, thereby reducing profits or causing losses for publicly funded stays [4]. Hospitals’ margins on publicly insured patients may also affect how hard they bargain with private insurers. A recent review by Frakt [5] found that cost-shifting from public to private insurance occurs, although it may be less than popularly imagined. Both studies imply that the Medicare and Medicaid payment levels may affect payment levels for other payers.

We wished to capture this within-hospital interaction in order to improve the accuracy of the PCR estimates. Our approach was an iterative estimation method proposed by Telser [6]. Equations are estimated individually and the errors are saved. Next, each payer equation is estimated a second time, with the first-stage errors of the other payer equations as new independent variables. The process may be repeated. There is no firm guidance available on when the estimates will converge, although Conniffe [7] suggests that it may occur as soon as the second stage. Here we adopt a two-stage model.

We assessed goodness of fit through model characteristics and by assessing the match of actual and predicted PCRs. The specific criteria include the predicted mean, mean absolute error (mean of the absolute value of the predicted errors), and root mean squared error (RMSE). RMSE is similar to mean absolute error but gives extra weight to larger deviations. Lower mean absolute error and lower RMSE indicate better fit.

### Variables

We represented casemix through payer-level averages within hospital of stay-level indicators for female gender, age group (1–7, 18–35, 36–45, 45–55, or 56 years and older), race/ethnicity (non-Hispanic White, non-Hispanic Black, Hispanic, and other or missing), and All-Patient Refined Diagnosis Related Group (APR-DRG) severity level. The severity levels indicate “the extent of physiologic decompensation or organ system loss of function” [8]. The levels range from 0–4, where 0 indicates no decompensation or loss of function and 4 indicates almost total decompensation or loss. Each value is represented by a separate indicator variable in the regression models. Each stands for the proportion of hospital stays during the study period that fell into that APR-DRG category.

Hospital classifications included average DRG weight, indicators for hospital designation as a critical-access facility, a rural referral center, a sole community provider, or a teaching hospital. We calculated average DRG weight within each payer across all discharges whose primary payer was the relevant payer category. It is weighted by discharge, rather than by person, as a single individual could have more than one hospital stay in our data. The critical-access designation was created to enhance the financial viability of small, isolated, rural, or otherwise necessary hospitals by requiring Medicare to pay them on a cost basis rather than prospectively [9]. Rural referral centers are rural hospitals with relatively high volume that treat a large number of complicated cases and transfers from small rural hospitals. The Herfindahl-Hirschman Index (HHI) captured competition in the hospital market. We defined the market as every hospital within 15 miles of the one at which a stay took place. Changing the radius likely would not affect the results [10].

As noted earlier, we limited our data to community hospitals in 10 states for which PCRs had been developed by Levit et al. [2]. The states were California, Florida, Massachusetts, Nevada, New Jersey, Virginia, West Virginia, Wisconsin, and two Northern states that did not give permission to be identified.

We extracted state-level variables from a variety of state sources that were likely to have an association with PCRs. Several variables capture state Medicaid program rules and funding and proxy the demand for Medicaid services. Others reflect general economic conditions of the state, which serve as proxies for the supply and demand for health care and the likelihood of being insured. The Medicaid data include the Medicaid income eligibility threshold as a proportion of the federal poverty level, for children and separately for adults; the Children’s Health Insurance Program (CHIP) uptake rate; and the Medicaid spending per capita. We included the number of federally qualified health centers (FQHCs) for every 1,000 nonelderly uninsured individuals in the state. FQHCs are safety-net providers who have access to special federal grants. The Medicare area wage index was included because hospitals in high- versus low-wage areas may have a different mix of labor and technology, which in turn may be reflected in charges. We hypothesized that the state’s fiscal and economic conditions will affect both supply and demand for health care and the level of private vs. public insurance. Included in the state’s financial health were the unemployment rate, the relative size of the state budget deficit, health spending per capita, per capita personal income, and the number of uninsured low-income residents under age 65. If there was no information pertaining to 2006—the year of the stays we studied—then we used the most recent year for which data were available. If that year was 2007 or later, then we used the data as a proxy for 2006 values.

The models for Medicare and Medicaid stays each contained four additional variables: the average number of Medicare (or Medicaid) stays and its square, and the average length of stay (ALOS) for Medicare (or Medicaid) stays, and its square. These variables reflect the hospital's scale and efficiency. Including the square enabled us to estimate whether payments relate to scale and efficiency without the assumption of linearity.

### Data

#### Healthcare cost and utilization project (HCUP)

Stay-level records were extracted from the HCUP State Inpatient Databases (SID), sponsored by the Agency for Healthcare Research and Quality (AHRQ). HCUP is one of the largest data sources on inpatient care in the United States, featuring records on more than 90 % of community hospital stays each year [11]. HCUP databases integrate the data collected by state governments, hospital associations, and private data organizations to create a national health care information resource of hospital, ambulatory surgery center, and emergency department data. In 2006 the SID captured most inpatient stays from all community hospitals in 46 states. The records include information on patient demographics, diagnoses, procedures, charges, payers and prices (payments), and hospital characteristics.

#### HCUP hospital market structure file

The HHI was derived from the HCUP Hospital Market Structure file. HHI equals the sum of the squares of the market shares for hospitals in the market.

#### State data

Table 1 lists the data sources for the state policies described earlier.

**Table 1:** Sources and definitions of analysis variables

Variable	Description
Age	Age group categories 0–17, 18–35, 36–45, 46–55, 56–64 years
Gender	Female
Race/ethnicity	Non-Hispanic White, Non-Hispanic Black, Hispanic, Other
APR-DRG Severity Index	Patients are classified into one of four severity-of-illness values based on clinical severity (minor, moderate, major, or extreme) according to clinical logic that includes interactions of multiple comorbidities, age, procedure, and principal diagnosis. Newborns and cases that cannot be classified are assigned a value of zero. All others receive a value from 1 to 4, where 4 indicates the greatest severity.
Teaching hospital status	Teaching status of the hospital (yes = 1, no = 0)
Critical access status	Hospital’s status as a critical-access hospital (yes = 1, no = 0). For criteria see https://www.cms.gov/CertificationandComplianc/04_CAHs.asp
Sole community provider status	Hospital’s status as the sole community provider (yes = 1, no = 0). For background information, see: https://www.cms.gov/Outreach-and-Education/Medicare-Learning-Network-MLN/MLNProducts/downloads//SoleCommHospfctsht508-09.pdf
Rural referral center status	Hospital’s status as a rural referral center (yes = 1, no = 0). For background information, see: http://www.cms.gov/Outreach-and-Education/Medicare-Learning-Network-MLN/MLNProducts/downloads//Rural_Referral_Center_Fact_Sheet.pdf
Hospital bed size	Number of inpatient beds, represented by four categories: <100, 100–199, 200–499, or 500 or more
Medicare average wage index	Regional wage index
Medicare inpatient days	Medicare average length of stay at the hospital in 2006
Medicare stays	Number of Medicare stays in 2006
Medicaid inpatient days	Medicaid average length of stay at the hospital in 2006
Medicaid stays	Total number of Medicaid stays at the hospital in 2006
Herfindahl-Hirschman Index (HHI) of hospital competition	The sum of the squared market shares of each hospital in the market. The market is defined as every hospital within 15 miles of the hospital at which a stay took place.
DRG Weight	Hospital-level average DRG weight across all included discharges for the payer.
FY2005 Deficit Dollars	FY 2005 deficit projection in millions of dollars (Center on Budget and Policy Priorities; FY 2004 General Fund data from NASBO, Fiscal Survey of the States, December 2003, Table A-3.)
2006 Per Capita Income by State	Income per capita by state in 2006 (Bureau of Economic Analysis, U.S. Department of Commerce, March 2012, Table SA1-3 Personal Income Summary)
Non-Elderly Uninsured Below the FPL	State number of non-elderly uninsured below the FPL. (Urban Institute and Kaiser Commission on Medicaid and the Uninsured estimates based on the Census Bureau’s March 2010 and 2011 Current Population Survey)
Working Parents Medicaid Eligibility	State Medicaid eligibility threshold for working parents as a proportion of the FPL. (Urban Institute and Kaiser Commission on Medicaid and the Uninsured estimates based on the Census Bureau’s March 2010 and 2011 Current Population Survey)
Medicaid per Capita	2006 Medicaid spending, total and per-capita spending (Public Policy Institute Analysis of Kaiser Family Foundation Data)
CHIP Rate	State Children’s Health Insurance Program. Medicaid/CHIP Participation Rates (Medicaid/CHIP Participation Rates 2011, see: http://www.insurekidsnow.gov/professionals/reports/index.html)
FQHC	Federally Qualified Health Centers (Service Delivery Sites), 2010 (see http://kff.org/other/state-indicator/total-fqhcs-service-delivery-sites/)

*Notes:* APR-DRG, All-Patient Refined Diagnosis Related Group; CHIP, Children’s Health Insurance Program; FPL, federal poverty level; FQHC, Federally Qualified Health Center; FY, fiscal year; NASBO, National Association of State Budget Officers

#### PCR values

We obtained from AHRQ the PCR values developed by Levit et al. [2]. They represent the dependent variables of the regression models.

### State fixed effects

Although our models control for a variety of state policies and characteristics, there could still be unobserved variation related to PCRs. We therefore estimated the same models a second time with state fixed effects in place of the state-level variables.

### Out-of-sample validation

To validate the PCR models, we arbitrarily chose one large state from each of three Census regions to exclude from a second round of regressions: California (West), Wisconsin (Midwest), and Florida (South). We then applied the resulting coefficients to stays in the omitted state and compared the estimated PCRs to the actual PCRs.

### Comparison to CCRs

HCUP cost-to-charge ratios (CCRs) may be used to estimate a hospital’s cost of producing care [12]. Although PCRs represent the payment rather than cost, they should be similar to CCRs because hospitals often experience low profit margins. If the PCRs and the CCR correlate highly, then the choice between them could become one of researcher convenience. Conversely, low correlations would suggest that studies using the CCR may be biased for one or more payer types, the cost and the payment may diverge substantially for certain payer types, or that PCRs cannot be estimated accurately for some states.

At the hospital level we calculated the Pearson correlation of each of the five estimated PCRs with the hospital CCR. The PCRs were the actual values for the 10 states from which they could be calculated directly (Levit et al. [2]) and predicted values from the second-stage regressions for the remaining 35 states. Because we believe that values below zero or greater than or equal to 1.0 represent outliers, in the correlation analysis we dropped hospitals for which *any* of the PCRs had those values. Results are shown with chi-square significance tests.

Analyses were carried out in SAS 9.2 (SAS Institute; Cary, NC) and Stata 11 (StataCorp; College Station, TX).

## Results

### Descriptive statistics

The contributing states had 1,144 hospitals in 2006 [2], about 20 % of all US community hospitals. After eliminating hospitals with missing or outlier PCRs, the count of hospitals by category was 1,110 for Medicare, 1,061 for Medicaid, 1,105 for private insurance, 899 for self-pay, and 868 for other insurance.

Table 2 presents descriptive statistics. The figures are the means of hospital-level figures. For example, .566 in the second column indicates that among hospitals with any Medicare patients, an average of 56.6 % of Medicare stays are for females.

**Table 2:** Hospital-level means by payer^a^

Characteristic	Medicare	Medicaid	Private	Self-Pay	Other
	mean (s.d.)	mean (s.d.)	mean (s.d.)	mean (s.d.)	mean (s.d.)
No. Obs.	1,110	1,061	1,105	899	868
Price-to-Charge Ratio					
Mean (SD)	.320 (.197)	.367 (.205)	.487 (.211)	.456 (.405)	.611 (.265)
Range	.098–.903	.176–.879	.197–.992	.193–.859	.268–.986
Average patient characteristics					
Gender (%)					
Female	.566 (.055)	.640 (.082)	.585 (.071)	.466 (.085)	.423 (.126)
Age group, years (%)					
0–17	.005 (.051)	.293 (.176)	.191 (.141)	.134 (.146)	.113 (.151)
18–35	.018 (.043)	.305 (.100)	.211 (.075)	.311 (.080)	.221 (.096)
36–45	.030 (.027)	.121 (.063)	.145 (.041)	.200 (.060)	.173 (.073)
46–55	.053 (.034)	.130 (.085)	.180 (.064)	.197 (.066)	.212 (.082)
56+	.894 (.106)	.151 (.129)	.272 (.134)	.159 (.084)	.281 (.157)
Race/ethnicity (%)					
Black	.087 (.134)	.156 (.177)	.086 (.131)	.119 (.145)	.109 (.125)
Hispanic	.079 (.146)	.219 (.236)	.107 (.163)	.186 (.198)	.136 (.166)
White	.709 (.301)	.471 (.296)	.655 (.295)	.552 (.278)	.615 (.276)
Other/missing race	.125 (.259)	.154 (.244)	.152 (.251)	.142 (.221)	.141 (.243)
Inpatient stay (%)					
Died	.042 (.040)	.013 (.037)	.014 (.041)	.015 (.034)	.023 (.053)
APR-DRG = 0	.000 (.001)	.000 (.003)	.000 (.002)	.001 (.003)	.000 (.003)
APR-DRG = 1	.195 (.075)	.505 (.154)	.539 (.120)	.507 (.108)	.485 (.136)
APR-DRG = 2	.453 (.065)	.340 (.089)	.334 (.071)	.361 (.071)	.357 (.079)
APR-DRG = 3	.282 (.071)	.124 (.073)	.100 (.055)	.104 (.051)	.125 (.070)
APR-DRG = 4	.070 (.052)	.031 (.030)	.026 (.048)	.027 (.020)	.033 (.029)
Hospital and market					
Critical access hospital (%)	.095 (.294)	.080 (.272)	.093 (.291)	.055 (.227)	.030 (.171)
Rural referral center (%)	.033 (.180)	.035 (.184)	.033 (.180)	.037 (.188)	.043 (.202)
Sole community provider (%)	.086 (.281)	.086 (.280)	.087 (.282)	.083 (.277)	.076 (.265)
Teaching hospital (%)	.294 (.456)	.311 (.463)	.300 (.459)	.320 (.467)	.324 (.468)
Herfindahl-Hirschman Index (HHI) *10	3.80 (3.23)	3.66 (3.14)	3.77 (3.21)	3.54 (3.08)	3.45 (2.99)
(HHI *10) squared	24.9 (34.9)	23.2 (33.6)	24.5 (34.6)	22.0 (32.8)	20.8 (31.4)
Average DRG weight	1.32 (.278)	.878 (.280)	1.03 (.310)	.975 (.234)	1.22 (.355)
Medicare disch./10,000	.419 (.408)	—	—	—	—
(Medicare disch./10,000)^2	.342 (.861)	—	—	—	—
Medicare ALOS	.640 (.467)	—	—	—	—
(Medicare ALOS/10)^2	.627 (2.17)	—	—	—	—
Medicaid disch/10,000	—	.205 (.269)	—	—	—
(Medicaid disch/10,000)^2	—	.114 (.450)	—	—	—
Medicaid ALOS/10	—	.601 (.946)	—	—	—
(Medicaid ALOS/10)^2	—	1.25 (17.2)	—	—	—
Wage index	1.07 (.178)	1.08 (.179)	1.08 (.180)	1.09 (.176)	1.08 (.492)
State					
2005 deficit ($1,000 s)	5,423 (6,211)	5,520 (6,193)	5,421 (6,184)	5,778 (6,251)	5,588 (6,290)
2006 per capita income ($1,000s)	41.07 (4.75)	41.3 (4.70)	41.1 (4.73)	41.6 (4.56)	41.3 (4.61)
Elderly under FPL (100,000s)	13.2 (11.1)	13.3 (11.1)	13.1 (11.1)	13.6 (11.2)	13.7 (11.1)
Medicaid eligibility threshold for children (% of FPL/100)	1.03 (.134)	1.03 (.139)	1.03 (.136)	1.04 (.150)	1.03 (.147)
Medicaid eligibility threshold for adults (% of FPL/100)	1.11 (1.34)	1.12 (.503)	1.13 (.508)	1.14 (.489)	1.08 (.492)
Medicaid per capita spending	1,137 (566)	1,153 (573)	1,149 (574)	1,162 (572)	1,119 (556)
CHIP enrollment rate	.851 (.061)	.853 (.059)	.852 (.061)	.853 (.059)	.848 (.062)
Number of FQHCs per 1,000 nonelderly uninsured	6.82 (3.20)	6.75 (3.10)	6.78 (3.16)	6.74 (3.07)	6.94 (3.21)

*Notes:* ALOS, average length of stay; APR-DRG, All-Patient Refined Diagnosis Related Group; CHIP, Children’s Health Insurance Program, DRG, diagnosis related group; FPL, federal poverty level; FQHC, Federally Qualified Health Center

^a^Figures are from the Medicare sample except for Medicaid ALOS and its square, which are from the Medicaid sample

The average PCR ranged from. 320 for Medicare stays to. 630 for those funded by other insurance. Within each payment category the PCRs had a range of 0.70 or greater, which represents wide variation across hospitals.

The first set of independent variables represents patient characteristics. Women represented the majority of insured stays. The age distribution varied considerably by payer type, which may reflect the rising illness rates across age groups, the effect (for Medicaid in particular) of eligibility based on pregnancy, and the preponderance of people over age 65 in Medicare based on age-related eligibility. Blacks and Hispanics were most common in the Medicaid and self-pay categories, which reflected a lower income and consequent lower likelihood of private insurance. There was considerable missingness in the race/ethnicity category, however, which limited our ability to draw firm conclusions. Only a small percentage of stays (1.3 to 4.2 %) ended in death. Relatively low APR-DRG Severity scores of 1 and 2 were most common, accounting for more than 70 % of stays.

Most hospital characteristics had similar frequencies across payers. Stays in critical access hospital were most common in stays funded by Medicare (9.5 %) or private insurance (9.3 %), although they were much less common for self-paid stays and those with other types of insurance. Only 3.3 to 4.3 % of stays across funders were in rural referral centers, reflecting their relatively small size. Sole community providers accounted for similar numbers of stays across payers as well, from 7.6 to 8.7 %. Stays in teaching hospitals were very common, representing 29.4 to 32.4 % of stays on average. The Herfindahl-Hirschman Index of hospital-market competition averaged between 3.45 and 3.81 by payer, with highest values for stays funded by Medicare and Medicaid. The average DRG weight varied considerably across payer groups, from .878 for Medicaid to 1.32 for Medicare, reflecting the difference in age distributions across payers.

Medicare stays averaged 4,190 per hospital with a length of 6.40 days. The average number of Medicaid stays was 2,050, and their average length was slightly shorter at 6.01 days.

The remaining variables were measured at the state level. The average state deficit was over $5.4 billion for each payer category, figures skewed upward by the presence of California. Per capita 2006 income averaged just over $41,000 across payer categories. There was moderate variation across states in the number of elderly uninsured under the federal poverty level (FPL), but relatively little across payers. The Medicaid eligibility threshold relative to the FPL was quite similar across payers for children’s eligibility (range 1.03 to 1.04) and for adults (range 1.03 to 1.13). Medicaid per capita spending ranged from $1,119 to $1,162 with moderate variation across states. The average CHIP enrollment rate among eligible individuals was 85 % for all payer groups. The mean number of FQHCs per 1,000 people ranged from 6.74 to 6.94 across payers.

### Generalized linear models of hospital payment-to-charge ratio by payer

Coefficients from the PCR regression models appear in Table 3. Each column represents a separate model, one for each payer type. These are second-stage results that include the error terms of the first stage as regressors. Each figure represents the percentage effect on the PCR of a one-unit increase in the independent variable, all else equal. Figures for age group, race/ethnicity, and APR-DRG severity index are interpreted relative to the omitted categories, respectively ages 56–65, White, and APR-DRG severity level 0 or 1.

**Table 3:** Generalized linear model of hospital payment-to-charge ratio by payer

Characteristic	Medicare	Medicaid	Private	Self-Pay	Other
Demographic (%)					
Female	.204	-.442	-.671***	.007	-.094
Age Group, years (%)					
0–17	1.26***	-.072	.497	-.246	-.328
18–35	.845***	.173	.974***	-.605*	−1.03*
36–45	2.78**	-.153	.057	-.563	−1.20*
46–55	-.431	-.123	.795	-.264	−1.01
Race/Ethnicity (%)					
Black	.225	.040	.037	-.180	.172
Hispanic	.262***	.158**	-.045	-.226*	.057
Other/missing race	-.071	.073	-.305**	-.003	.304***
Inpatient Stay (%)					
Died	2.03*	3.59**	.696	−3.05	.238
APR-DRG = 2	.044	.209	.467**	-.103	-.292
APR-DRG = 3	-.472*	.776**	.816	.038	−1.68*
APR-DRG = 4	-.539	−4.71***	−2.04	-.034	-.955
Hospital And Market					
Critical access hospital	.572***	.502***	.153	-.103	-.014
Rural referral center	.070**	.109**	.026	-.038	.062
Sole community provider	.193***	.096	.219***	-.034	.033
Teaching hospital	.180***	.180***	.074**	-.059	-.114*
Herfindahl-Hirschman Index (HHI)	.019***	.077***	.101***	-.004	-.161*
HHI squared	.001***	-.004***	-.005***	.001	.013*
Average DRG weight	.133	.058	.011	.177*	.071
Medicare disch./10,000	.005	—	—	—	—
(Medicare disch./10,000)^2	-.007	—	—	—	—
Medicare ALOS/10	-.385	—	—	—	—
(Medicare ALOS/10)^2	.142	—	—	—	—
Medicaid disch/10,000	—	-.052	—	—	—
(Medicaid disch/10,000)^2	—	.015	—	—	—
Medicaid ALOS/10	—	.065***	—	—	—
(Medicaid ALOS/10)^2	—	.003***	—	—	—
Wage index	-.452***	-.464***	.056	-.093	-.177
State					
2005 deficit ($)	-.033*	-.021***	-.019*	-.039***	-.040***
2006 per capita income ($)	.018	.039***	.000	.084***	.033***
Elderly under FPL (100,000 s)	.000	-.000	.000	.000***	.000
Medicaid eligibility threshold for children (% of FPL)	-.686**	−1.41***	-.754***	−3.97***	−2.21***
Medicaid eligibility threshold for working parents (% of FPL)	.425***	.194***	.303	.563***	.195***
Medicaid per capita spending ($)	.236***	.289***	.000***	-.181***	-.213***
CHIP enrollment rate	−8.34***	−7.86***	−6.99***	−8.17***	.627
Number of FQHCs per 1,000 nonelderly uninsured	-.000***	-.000***	-.166***	-.000***	-.037***
First-Stage Errors					
Medicare PCR	---	1.30***	.582***	.095	.196
Medicaid PCR	1.37***	---	.224	.151	-.012
Private PCR	.598***	.109	---	-.236	-.025
Self-Pay PCR	-.012	-.022	-.226	---	-.092
Other PCR	-.007	-.021	-.058**	-.089	---
Intercept	6.71***	6.53	6.57	8.26	1.61
Number of Observations	852	856	854	778	817

*Notes:* Figure are exponentiated coefficients. ALOS, average length of stay; APR-DRG, All-Patient Refined Diagnosis Related Group; CHIP, Children’s Health Insurance Program; disch., stay; DRG, diagnosis related group, FPL, federal poverty level; FQHC, federally qualified health center; PCR, payment-to-charge ratio

*p < .05, **p < .01, ***p < .001

Average demographic characteristics among a hospital’s inpatients were significant predictors of PCRs in all insurance categories. Female gender was only significant for private insurance, where it had a large, negative coefficient. Age groups were significant for all categories except Medicaid. Large, positive coefficients for Medicare recipients under age 65 may reflect the difference between the omitted category (age 65 or older), which includes people of all health levels, and the younger age groups, whose eligibility indicates chronic and substantial disability. Either Hispanic ethnicity or other/missing race was statistically significant in each model, while Black race was always insignificant.

Hospital-level proportions of the two *ex post* severity measures—death during the inpatient stay and APR-DRG severity level—were significantly related to PCRs for Medicare, Medicaid, privately funded, and other-funded stays, but not to stays self-paid by patients. Hospitals with higher proportions of deaths had higher PCRs while those with higher proportions of high-APR-DRG stays (patients with higher severity) had lower PCRs.

The four special hospital designations were significantly related to every PCR category except self-pay. All of the variables were associated with higher Medicare PCRs, three with Medicaid and private insurance, and one with other. Being a critical-access hospital was associated with higher PCRs for Medicare and Medicaid, which implies that the cost-based payments they receive are closer to their charged amounts than are standard prospective payments. Rural referral status was significantly related to higher Medicare and Medicaid PCRs as well. Sole community provider status was also associated with higher PCRs for stays paid by Medicare and private insurance but not for Medicaid. Teaching hospital status was associated with significantly greater PCRs for stays paid by Medicare, Medicaid, and private insurance, but lower PCRs for those paid by other insurance.

The HHI based on 15-mile radius had a positive and significant association with the PCR for all payer categories except self-pay. For private insurance it follows the standard economic reasoning that hospitals in more concentrated markets use their greater bargaining power to extract higher payments. For other payers rates are not set by hospital-level negotiation, and so the relation of HHI to PCRs most likely reflects geographic correlation with causal factors rather than direct causality. The total impact is relatively small regardless. For private payers, the combined effect of an increase in HHI of 10 percentage points, calculated over an HHI range of 20 to 50 percent, is to increase the PCR by 2.0 to 4.4 %.

Average DRG weight was significantly related to PCR only for self-pay stays, where it had a small positive coefficient. The number of Medicare and Medicaid stays were all insignificant. Medicare average length of stay (ALOS) was insignificant as well, while greater Medicaid ALOS was correlated significantly with higher Medicaid PCRs. Higher area wages were significantly related to lower PCRs for Medicare and Medicaid, which implies that higher wages lead to higher charges that are not fully captured in wage adjustments made in these federal programs. Alternatively it could reflect higher charges for other reasons in areas that also have higher wages.

The state characteristics in the models were strongly associated with several PCRs. Lower PCRs were most often associated with worse economic conditions (higher deficits, lower income) and a more generous (higher) Medicaid eligibility threshold for children. Conversely, more generous eligibility thresholds for working-age parents were associated with higher PCRs for four of the five insurance categories. Higher Medicaid spending per capita was positively related to PCRs for Medicaid, Medicaid, and private insurance but lower PCRs for self-pay and other insurance. Higher CHIP enrollment rates were associated with sharply lower PCRs for all categories except other insurance. A greater number of FQHCs were significantly related to lower PCRs in all models.

At least one of the first-stage error terms was significant in the Medicare, Medicaid, and private insurance models. In two cases the relationship was reflected in mutual significance of one’s error in the other’s model: Medicare and Medicaid, and Medicare and private insurance. The insignificant coefficients were much smaller and were as likely to be negative as positive.

### Alternative models with state fixed effects

An alternative set of models replaced the state-level variables with state fixed effects. Of the 9 included state effects—California was the excluded category—from 3 to 7 were significant at the 99 % confidence level in each regression. They produced results (not shown) similar to those of the main models but with slightly worse corrected values of the corrected Akaike Information Criterion [13], a measure of overall goodness of fit. We therefore conclude that the main models are doing a sufficient job of capturing state-level variation relevant to PCRs.

### Out-of-sample validation

Table 4 resents results of the out-of-sample validation exercise. We estimated the regression models for each PCR a total of three times, each time omitting one state. We then estimated the RMSE of the PCRs for the included states and, based on out-of-sample estimation, for the omitted states.

**Table 4:** Out-of-sample validation: RMSEs by payer and omitted state

Dependent variable	RMSE for included states	RMSE for omitted state
Omitting California		
PCR 1 Medicare	.174	.177
PCR 2 Medicaid	.186	.287
PCR 3 Private	.194	.339
PCR 4 Self-Pay	.226	.608
PCR 5 Other	.224	.702
Omitting Wisconsin		
PCR 1 Medicare	.166	.269
PCR 2 Medicaid	.191	.203
PCR 3 Private	.180	.454
PCR 4 Self-Pay	.236	.452
PCR 5 Other	.271	.594
Omitting Florida		
PCR 1 Medicare	.173	.269
PCR 2 Medicaid	.196	.116
PCR 3 Private	.210	.311
PCR 4 Self-Pay	.231	.538
PCR 5 Other	.273	.900

*Notes:* RMSE, root mean squared error

Out-of-sample RMSE values were lowest for Medicare (range .177 to .269) and Medicaid (.116 to .287) and highest for self-pay and other categories (.452 to .900). In-sample RMSE was lower than the out-of-sample RMSE in most cases, as expected.

The results varied notably by omitted state. For privately funded stays, for example, the RMSE for the omitted state was .339 for California, .454 for Wisconsin, and .311 for Florida. The average PCR for privately funded stays across all states was .487. The out-of-sample RMSEs for privately funded stays therefore represent 69.6 %, 93.2 %, and 63.9 % of the mean, respectively.

### Correlations between PCRs and CCRs

Table 5 presents descriptive statistics and Pearson correlations of the HCUP CCRs and the five PCRs. The 838 hospitals represented here had *all* PCRs in the range of 0 to 1; this is more restrictive than the regressions, which require only that the PCR serving as the dependent variable fall in that range. We found that CCRs were highly correlated with estimated PCRs for Medicare, Medicaid, and private insurance (range 0.75–0.79, *p <* .01) but were uncorrelated with PCRs for self-pay and other insurance. Estimated PCRs for Medicare, Medicaid, and private insurance were highly correlated as well (range 0.62–0.77, all *p* < .01). The self-pay PCRs were significantly correlated with the PCRs for Medicare, Medicaid, and private insurance, although the correlations were low (range −0.10 to 0.21, all *p <* .01). The PCRs for other payers were likewise correlated significantly with Medicaid and self-pay PCRs but with low correlations (range 0.08 to 0.24, both *p <* .05).

**Table 5:** Pearson correlation coefficients of CCRs and PCRs (*N =* 838)

Measure	CCR	PCR1	PCR2	PCR3	PCR4	PCR5
CCR	1.00	0.78**	0.76**	0.75**	0.06	0.00
PCR Medicare		1.00	0.77**	0.69 **	0.12 **	0.00
PCR Medicaid			1.00	0.62 **	0.21 **	0.08 *
PCR Private				1.00	−0.10 **	−0.06
PCR Self-Pay					1.00	0.24 **
PCR Other						1.00

*p < .05, **p < .01

Note: sample limited to hospitals for which all PCR values fall between 0 and 1

## Discussion

Levit and colleagues [2] showed that payments could be estimated from charges and other hospital information in 10 States. They estimated payment-to-charge ratios for five types of payers for each facility. If PCRs are to be used widely for research, it must be feasible to calculate or estimate their values for a larger number of states. The remaining states do not provide enough financial detail for exact PCRs to be derived. We therefore investigated whether PCRs can be estimated with reasonable accuracy based on characteristics of the state and of each hospital and its patients. We find that the estimates have moderate accuracy for the most common payer categories of Medicare, Medicaid, and private insurance. Although Medicare and Medicaid fee-for-service payments can be estimated by other means, they will not match the PCRs for those payments (Levit et al. [2]). PCRs may represent the best method currently available for predicting what hospitals receive from Medicare, Medicaid, and privately insured patients.

The estimated prediction errors of the PCRs were not small. The relative errors (RMSE divided by mean PCR) from Table 4 range from was 55 % to 84 % for Medicare, according to which state was omitted; from 32 % to 78 % for Medicaid; from 64 % to 93 % for private insurance; from 99 % to 133 % for other insurance; and from 97 % to 147 % for self-pay. If a hospital charged $10,000 for a Medicaid stay and received $4,000, it would represent a true PCR of 40 %. An absolute prediction error of ± 50 % would yield predicted payments ranging from $2,000 to $6,000. Whether this range is sufficiently small will depend on the user’s needs. We are not aware of studies of bias from using the CCR or some other alternative, and so the relative size of the PCR prediction error remains unknown.

We acknowledge potential endogeneity between PCRs and the predictive characteristics. For example, we use demographic characteristics specific to each payer and hospital. Over the long run hospitals may react to PCRs by selectively attempting to build business among patients with certain conditions. If successful, these efforts would likely affect the demographic mix. Market share could be endogenous as well if hospitals choose to locate in high-margin regions. We partially control for this through state-level variables, but there may be longer-term patterns that a time-series analysis could reveal. The HCUP Hospital Market Structure File is created only every three years, however, and interpolation of intermediate years would be arbitrary.

### PCRs versus alternative estimators

The Medicare PCR represents an alternative to the Medicare CCR and to the Medicare payment estimated by pricer software. Levit et al. [2] explains the development of the PCR in detail, noting that the average price of hospitals stays in the 10-state sample is considerably different under the Medicare pricer ($9,850) and the Medicare PCR ($8,418). This difference is enough to suggest that simply using the pricer output could lead to a substantially different answer to most research questions.

Medicaid fee-for-service payments for certain stays may be calculated based on publicly available payment rules. (Hospital-level bonuses or penalties raise the additional issue of how to attribute those to individual stays.) Fee-for-service (FFS) equivalent payments could likewise be estimated for stays under capitated managed-care plans if a state has a public FFS payment formula. Gathering the information, particularly interstate variations in Medicaid payment and coverage, could be quite burdensome for a regional or national study. For Medicaid-funded stays within one or a few states, though, this approach could be preferable.

PCRs may be the only option beyond CCRs for estimating prices for privately insured and self-paid stays, and the few funded by other types of insurance. Little or no information is publicly available on payments from those insurance sources, and almost by construction there is no information on self-paid stays.

We found that CCRs were highly correlated with PCRs for Medicare, Medicaid, and private insurance. A high correlation makes sense given the relatively low profit margins of hospitals; a low profit margin indicates that payments are similar to costs on average. CCRs did not correlate well with PCRs for self-pay and other insurance, however. This could reflect an inherent lack of correlation or variation across hospitals in our ability to accurately estimate PCRs for those categories of payers. Regardless, correlation does not imply a lack of bias nor the relative size of any bias in CCRs or PCRs, but merely that the CCR and PCR values tend to move in tandem across hospitals.

### Uses of PCRs

PCRs can assist researchers and policymakers in several ways. The most obvious is to observe how payments vary by primary payer. They can be used to understand differences in payments across geographic regions, at any level of geographic specificity, and to see how those react to policies at the state and federal level. A study by White [14] analyzed how hospitals respond to Medicare payment cuts; PCRs would provide an alternative and straightforward method for measuring Medicare payments, and it would allow estimation of the impact of Medicare payment changes on payments by private insurers. PCRs can help public policymakers to better understand hospitals’ responses to differential payment amounts for similar services across payers. PCRs represent an alternative to proprietary databases for obtaining estimated payments for privately insured stays. They also provide insight into payments received for uninsured stays, a category not found in claims databases because no insurance claim was filed.

We see three options for researchers considering use of PCRs. One is to use the parameter estimates from our tables with relevant hospital and state characteristics to develop PCRs for additional states. This approach would be useful for developing payment metrics for broad analyses. If greater precision is needed, then it may be advisable instead to use the estimates from Levit et al. [2] in the original 10 states. A third option is to use CCRs as a rough approximation of PCRs. The two have fairly high correlations for major payer categories. With the caveats noted above, using CCRs may be sufficient for comparisons within a small group of hospitals.

## Conclusion

Hospital payments for stays funded by Medicare, Medicaid, and private insurance can be predicted with moderate accuracy for states where this information is not provided. Together with the directly calculated PCRs available from Levit et al. [2], researchers can now obtain payment estimates for community hospital stays from major payer groups.

The results illustrate the wide range of hospital payment-to-charge ratios by payer and the considerable variation in these ratios across states. Because the prediction errors are large for predicted PCRs of self-pay and other insurance, better models will be needed before they can be estimated with acceptable accuracy.
